# Patients’ Journey and Burden of Disease Among Patients with Psoriatic Arthritis in Greece: A Cross-Sectional Survey

**DOI:** 10.31138/mjr.0210250.arg

**Published:** 2026-06-09

**Authors:** Garyfallia Stefanou, Dimitra I. Lampropoulou, Katerina Lioliou, George Gounelas, Katerina Koutsogianni, Vasileios Kountouris, Michael Feretos, Georgia Kourlaba

**Affiliations:** 1 ECONCARE LP, Athens, Greece;; 2 PanHellenic Federation Reumazin, Thessaloniki, Greece;; 3 UCB Pharma, Athens, Greece;; 4 Department of Nursing, University of Peloponnese, Tripoli, Greece

**Keywords:** psoriatic arthritis, surveys and questionnaires, patient journey, humanistic and economic burden, diagnostic delays, health-related quality of life (HRQoL)

## Abstract

**Objective/Aim:**

This study aims to explore the patients’ journey from symptom onset to diagnosis and treatment and evaluate the humanistic and economic burden of Psoriatic Arthritis (PsA) in Greece.

**Methods:**

A cross-sectional online survey was conducted with patients with PsA over 18 years old, members of the Greek patients’ association “Reumazin”. Data were collected through a structured questionnaire covering socio-demographics, medical history, patients’ journey, health-related quality of life (HRQoL) using the Psoriatic Arthritis Quality of Life (PsAQoL) questionnaire, work productivity impairment, treatments, healthcare utilisation, and expenditures over the past 12 months. The economic burden included out-of-pocket expenses and indirect cost.

**Results:**

A total of 148 patients with PsA participated, (70% female, median age 56 years). Median times from symptoms onset to rheumatologist visit, diagnosis, and treatment initiation were 11.5, 12.8, and 15.3 months, respectively. Most common initial symptoms were skin rash/psoriasis (77%) and joint pain (45%). Initial specialists visited included dermatologists (42%) and orthopaedics (21%), with 47% of patients initially misdiagnosed. The median PsAQoL score was 9.0 (Q1: 4.0, Q3: 13.0). Among employed participants (47%), median overall work impairment was 30%, and activity impairment was 40%. Mean annual out-of-pocket expenses were €609 (95% CI: €535 – €704), driven by treatment costs (70%). Indirect cost was €3,723 (95% CI: €2,906 – €4,859) with presenteeism accounting for 82%.

**Conclusion:**

This study highlights diagnostic delays and the humanistic and economic burdens of PsA in Greece, providing insights into unmet needs to support healthcare providers and policymakers in improving patient care and resource allocation.

## INTRODUCTION

Psoriatic arthritis (PsA) is a chronic inflammatory condition that shares many clinical features with other spondyloarthropathies and rheumatoid arthritis.^1^ PsA is characterised by musculoskeletal manifestations, such as back pain, peripheral arthritis, enthesitis, and dactylitis, as well as extra-articular features, including uveitis, inflammatory bowel disease, and skin psoriasis. The most common clinical symptoms are oligoarthritis or polyarthritis and enthesitis.^2,3^ PsA affects 0.05% to 0.25% of the general population.^4^ In Europe and North America, its prevalence ranges between 20 and 420 per 100,000 people, with an incidence between 3 and 23.1 per 100,000 people.^5^ According to a 2019 meta-analysis, the overall prevalence of PsA in patients with psoriasis is 20%, rising to 25% in those with moderate to severe psoriasis. In Greece, prevalence of PsA among patients with psoriasis is estimated at 30%.^6,7^

Physical trauma, obesity, alcohol consumption, smoking, and depression are linked to PsA onset.^8^ Individuals with PsA are at greater risk of chronic and potentially life-threatening comorbidities, including cardiovascular diseases, metabolic syndrome, diabetes mellitus, osteoporosis, and hyperlipidaemia.^9^ A significant challenge in managing PsA is frequent diagnostic delays, often exceeding two years, which increase the clinical and economic burden for patients and healthcare systems.^10– 12^ Even a 6-month delay from symptom onset to the first visit to the rheumatologist leads to worse outcomes for patients with PsA.^13^ Emerging evidence suggests that a *window of opportunity* may exist in PsA, meaning that shorter delays from symptom onset to diagnosis (particularly within one year) are associated with improved clinical outcomes, such as higher rates of remission.^14,15^ Over the past decade, PsA management has improved with novel biologic and targeted disease-modifying anti-rheumatic drugs (DMARDs), but prolonged diagnostic delays still result in poorer outcomes and functional impairment.^10,15,16^ Clinician-related factors, such as misdiagnosis and lack of disease-specific biomarkers, contribute to these delays.^17^ Consequently, many patients already have functional limitations by the time they are diagnosed.^18^

In Greece, real-world data on patient experiences with PsA, especially regarding diagnostic delays, are limited.^19–22^ There is a scarcity of data on annual direct cost from the payer’s perspective; recently, Tsifetaki et al. conducted a cost-of-illness analysis, revealing that the total annual direct cost per patient from the payer perspective was €8,097 ± €6,802.^23^ Additionally, to the best of our knowledge, no data are available for the out-of-pocket expenses and the indirect cost per patient. This study aims to explore the patients’ journey from symptom onset to diagnosis and treatment and to evaluate the humanistic burden, work impairment, and out-of-pocket expenses of patients with PsA in Greece.

## MATERIALS AND METHODS

A cross-sectional online survey was conducted from September 2023 to February 2024. Eligible to participate in the study were patients diagnosed with PsA, adults (≥18 years old), members of the Greek patients’ association “Reumazin” regardless of current treatment regimen and disease severity, who spoke and read Greek and provided signed consent form. Members were reached via email by the administration staff of the patients’ association. Before initiating the questionnaire, participants were required to review an electronic informed consent form detailing the study objectives, voluntary participation, data anonymity and confidentiality, and the right to withdraw at any time. Consent was confirmed electronically confirmation prior to survey access. The protocol of the study was approved by the management board of “Reumazin” in July 2023, while the study adhered to the ethical principles of the Helsinki Declaration. The design and reporting of this study followed the proposed guidelines for survey-based research of Zimba and Gasparyan.^24^

Sample size estimation for “Time from symptom onset to rheumatologist visit” required 131 participants based on the methods of Johnston et al. (2019), assuming 1.00 years (Q1–Q3: 0.5–2), 95% confidence, and 20% precision. (13,25) Accounting for a 10% non-response rate, the minimum required sample size was 144 participants.

### Data collection

Data were collected through a structured questionnaire hosted on Google Forms and distributed to eligible patients by the “Reumazin” association (the English version is provided in the Supplementary Material). Prior to questionnaire development, key domains were defined based on a literature review and expert consultation, with patient representatives contributing to refinement of wording and ensuring content relevance. The preliminary version of the questionnaire was piloted with 10 patients to evaluate clarity and feasibility, leading to minor adjustments based on their input. Subsequently, an expert panel comprising two rheumatologists, two general practitioners, and two patient representatives reviewed the instrument and confirmed its adequacy and comprehensiveness in addressing the study objectives.

The questionnaire gathered data on socio-demographic characteristics included sex, age, residence, education level, current employment status, marital status, current annual income, weight, height, and smoking status. Medical history covered comorbidities and family history of PsA. The patient’s experience with PsA was assessed with a selection of 10 questions of a previously published, US web-based survey, covering symptoms, time to seek care, year of diagnosis, time to diagnosis, initial physician specialty, misdiagnosis, time to rheumatologist visit, time to first treatment, initial medications, and current physician specialty.^10^ Health-related quality of life (HRQoL) was measured using the Psoriatic Arthritis Quality of Life (PsAQoL) questionnaire for which a certified translated version of the questionnaire is available in Greek. PsAQoL includes 20 items, each requiring a ‘true’ or ‘not true’ response, and leading to a total score ranging from 0 to 20, where higher scores indicate poorer HRQoL.^26,27^

Work productivity impairments were evaluated using the Work Productivity and Activity Impairment (WPAI) questionnaire, generating scores for absenteeism, presenteeism, overall work productivity loss, and activity impairment outside work, with higher scores indicating greater impairment.^28^ Absenteeism and presenteeism costs were derived using the corresponding items from the WPAI questionnaire. Weekly absenteeism cost was calculated by multiplying hours lost from work with mean hourly earnings in Greece (€21,297 GDP per capita in 2023 as provided by the International Monetary Fund (IMF) / 245 working days / 8 hours). Weekly presenteeism cost was estimated by multiplying affected hours with reduced productivity percentage and mean hourly earnings. Annual absenteeism and presenteeism costs were weekly costs multiplied by 49 working weeks.

Participants were also asked about current treatments, including topical treatments, non-steroidal anti-inflammatory drugs (NSAIDs), corticosteroids, conventional DMARDs (csDMARDs), biologic DMARDs (bDMARDs), and targeted synthetic DMARDs (tsDMARDs).

Lastly, patients were asked to report their healthcare utilisation and out-of-pocket expenses over the past 12 months. This included information on the number and duration of hospitalisations, frequency of visits to private doctors or healthcare units, ongoing treatments, and the total out-of-pocket costs associated with these healthcare services due to their condition.

### Statistical analysis

Continuous variables are presented using means and standard deviation (SD) or medians and 1st and 3rd quartiles (Q1 – Q3). Categorical variables are presented using absolute and relative frequencies. Costs are presented with means and 95% confidence intervals (CI). As cost data tend to be truncated at zero and do not usually follow normal distributions (skewed because they were bounded by zero and because there were often small numbers of very costly cases), the 95% CI for mean costs were derived using 5,000 non-parametric bootstrap resamples, performed by resampling the data with replacement.

For time from symptoms’ onset to treatment initiation, the proportionality of hazards assumption between the categories of possible associated factors was assessed graphically with log-log plots and with the comparison of the fit of Kaplan-Maier survival curves with the predicted survival from the Cox model, and statistically on the basis of Schoenfeld residuals after fitting a Cox model. Univariate parametric Cox models were applied to determine associations with time from symptoms’ onset to treatment initiation. All factors with a p-value less than 0.05 were included in a multivariate model.

Univariate and multivariate generalised linear models (GLMs) with Gaussian family and identity link function were applied to identify associations with HRQoL. To determine factors associated with economic burden [out-of-pocket expenses, annual indirect cost (absenteeism and presenteeism costs)] we applied GLMs with gamma family and a log link function, which best fit the expenditure data according to the Box-Cox test and the modified Park test. Baseline demographic, lifestyle, anthropometric and clinical characteristics of patients were evaluated for potential associations; those with a p-value less than 0.15 were included in the multivariate models. The significance level was set to a=0.05.

All analyses were performed using STATA software (version 17.0, 2021, STATA Corp).

## RESULTS

### Patient characteristics

Out of 294 members of ‘Reumazin’ with PsA, 163 more active and informed members were contacted, and 148 participated in the survey, resulting in a completion rate of 90.8%. Of these, 70% were female, with a median age of 56.0 years (Q1: 45.0, Q3: 66.5). Additionally, 85% of the participants reported having at least one comorbidity, with hypercholesterolemia (34%) and osteoporosis (33%) being the most common. At the time of survey completion, 95% of participants were receiving treatment for PsA; 61% were treated with b/tsDMARDs, either as monotherapy or in combination with csDMARDs, NSAIDs, corticosteroids, and/or topical treatments. Most commonly used biologic agents were the TNF-α inhibitors (61% among those under b/tsDMARDs). Conventional DMARDs either alone or in combination with NSAIDs, corticosteroids, and/or topical treatments were used by 29% of the participants (**Table 1**).

**Table 1.** Demographics, clinical profile, and treatment of study participants (N=148).

**Demographics, Clinical profile, and Treatment**	**N=148**
**Demographics – General profile**	
**Sex, n (%)**	
Female	104 (70.3%)
Male	44 (29.7%)
**Age, years**	
Median (Q1 – Q3)	56.0 (45.0 – 66.5)
**Residence, n (%)**	
Urban area	113 (76.3%)
Suburban / Rural area	35 (23.7%)
BMI, n (%)^*^	
Underweight / Normal range	57 (38.5%)
Overweight	56 (37.8%)
Obese	35 (23.6%)
**Marital status, n (%)**	**N=146**
Unmarried / Divorced / Widow(er)	53 (36.3%)
Married / cohabitation	93 (63.7%)
**Occupational status, n (%)**	
Freelancer / self-employed	31 (20.9%)
Employee	36 (24.3%)
Unemployed	8 (5.4%)
Retired	58 (39.2%)
Student	1 (0.7%)
Household	13 (8.8%)
Other: Farmer	1 (0.7%)
**Education, n (%)**	**N=147**
Primary school / Lower secondary education / Upper secondary education	86 (58.5%)
Bachelor’s degree	45 (30.6%)
Master’s/Doctoral degree	16 (10.9%)
**Annual household income, n (%)**	**N=126**
<10,000 €	27 (21.4%)
10,000 € – 20,000 €	58 (46.0%)
20,000 € – 30,000 €	29 (23.0%)
> 30,000 €	12 (9.5%)
**Smoking habits, n (%)**	
Smoker	39 (26.4%)
Former smoker	40 (27.0%)
No smoker	69 (46.6%)
*Medical Profile*	
**Comorbidities, n (%)**	**N=137**
None	21 (15.3%)
One	40 (29.2%)
Two	27 (19.7%)
Three or more	49 (35.8%)
Comorbidities, n (%) ^**^	**N=137**
Hypercholesterolemia	46 (33.6%)
Osteoporosis	45 (32.8%)
Arterial hypertension	41 (29.9%)
Fibromyalgia	33 (24.1%)
Depression or anxiety disorder	33 (24.1%)
Diabetes Mellitus	21 (15.3%)
Asthma	19 (13.9%)
Coronary heart disease (heart failure, arrhythmia, heart valve problems)	15 (11.0%)
Ocular disease of autoimmune aetiology (Uveitis, Keratitis, Blepharitis, Conjunctivitis, Episcleritis)	11 (8.0%)
Crohn's disease or ulcerative colitis	6 (4.4%)
Non-alcoholic fatty liver	6 (4.4%)
Cerebrovascular diseases (Stroke, Peripheral artery disease, Aortic Valve Disease)	5 (3.7%)
Other	4 (2.8%)
**Family history of PsA, n (%)**	**N=106**
Yes	71 (67.0%)
No	35 (33.0%)
***Treatment of PsA & Treatment perspectives***	
**Treatment combinations, n (%)**	**N=147**
Only b/tsDMARDs	43 (29.3%)
b/tsDMARDs combinations^1^	46 (31.3%)
Only csDMARDs	17 (11.6%)
csDMARDs combinations (excluding b/tsDMARDs)^2^	25 (17.0%)
Other^3^	8 (5.4%)
No treatment	8 (5.4%)
On-going treatment among those treated, n (%) ^1**^	N=139
Biologic agents (bDMARDs) and/or JAKs (tsDMARDs)	89 (64.0%)
csDMARDs [Methotrexate, Leflunomide (Arava®), Sulfasalazine (Azulfidine®)]	66 (47.5%)
Oral corticosteroid	40 (28.8%)
Topical treatments (creams, ointments)	27 (19.4%)
NSAIDs	8 (5.8%)
Cortisone via intra-articular/local injection	6 (4.3%)
Phosphodiesterase-4 inhibitor (Apremilast (Otezla®))	2 (1.4%)
Other: Painkillers	1 (0.7%)
Agents among those under b/tsDMARDs, n (%) ^**^	**N=89**
TNF-α inhibitor [Adalimumab (Humira®), Certolizumab Pegol (Cimzia®), Etanercept (Enbrel®), Golimumab (Simponi®), Infliximab (Remicade®/Remsima®/Inflectra®)]	54 (60.7%)
Interleukin -12/23 inhibitor [Ustekinumab (Stelara®)]	10 (11.2%)
Interleukin-23 inhibitor guselkumab [(Tremfya®, Risankizumab (Skyrizi®)]	1 (1.1%)
Interleukin -17 inhibitor [Ixekizumab (Taltz®), Secukinumab (Cosentyx®)]	15 (16.9%)
Co-stimulation inhibitor [Abatacept (Orencia®)]	9 (10.1%)
JAK kinase inhibitor [Tofacitinib (Xeljanz), Upadacitinib (Rinvoq®)	1 (1.1%)

1 with csDMARDs, NSAIDs, corticosteroids, and/or topical treatments;

2 with NSAIDs, corticosteroids, and/or topical treatments;

3 NSAIDs, corticosteroids, topical treatments, and/or painkillers.

* BMI was categorised as underweight (<18.5), normal range (18.5 to <25), overweight (25 to <30), and obese (≥30), according to standard World Health Organisation classifications.

** Each participant could select more than one option.

cs/b/ts DMARD: conventional/biologic/targeted synthetic disease-modifying antirheumatic drug; NSAIDs: Non-Steroidal Anti-Inflammatory Drugs; Q: quartile; TNF-α: Tumour Necrosis Factor alpha.

### Diagnosis and patients’ journey

Among the participants, 146 reported experiencing at least one symptom that prompted them to seek medical care. The most common symptom was skin rash/psoriasis (77%), followed by joint pain (45%). The median age at PsA diagnosis was 41.0 years (Q1: 33.0, Q3: 50.0). Initially, the most frequently consulted specialists were dermatologists (42%) and orthopaedics (21%). At the time of data collection, 95% of participants were seeing a rheumatologist. Additionally, 47% of participants had been initially misdiagnosed. While 53% could not recall their first medication for PsA, among those who did remember (n=79), the first treatments reported included conventional DMARDs (41%), corticosteroids (35%), b/tsDMARDs (16%), cyclosporine (5%), and painkillers (3%). The median (Q1 – Q3) times from the onset of the first symptom to the initial visit with a rheumatologist, to diagnosis, and to treatment initiation were 11.5 (5.0 – 20.5) months, 12.8 (6.0 – 18.5) months, and 15.3 (9.0 – 23.5) months, respectively (**Table 2**).

**Table 2.** Patients’ journey from symptoms onset to treatment initiation.

**Pathway to PsA diagnosis**	**Ν=148**
**Symptoms that led you to seek medical care, n (%)** ^*^	**Ν=146**
Skin Rash/Psoriasis	112 (76.7%)
Joint Pain	66 (45.2%)
Swollen Joints	50 (34.2%)
Back Pain	41 (28.1%)
Fatigue	41 (28.1%)
Stiffness	35 (24.0%)
Low Back Pain	34 (23.3%)
Tendon/Ligament Pain	26 (17.8%)
Neck Pain	24 (16.4%)
Nail problems	24 (16.4%)
Sleep Problems	24 (16.4%)
Difficulty Walking	20 (13.7%)
Eye Problems	13 (8.9%)
Chest Pain	5 (3.4%)
Shortness of Breath	5 (3.4%)
Pelvic Pain	1 (0.7%)
**Specialty of the physician you first visited, n (%)**	**N=145**
Dermatologist	61 (42.1%)
Orthopaedist	30 (20.7%)
Pathologist/General Practitioner	27 (18.6%)
Rheumatologist	24 (16.6%)
Gastroenterologist	1 (0.7%)
Neurologist	1 (0.7%)
Pneumonologist	1 (0.7%)
**What specialty has the physician currently seeing you for PsA, n (%)**	
Dermatologist	6 (4.1%)
Orthopaedist	1 (0.7%)
Pathologist/General Practitioner	1 (0.7%)
Rheumatologist	140 (94.6%)
**Time Intervals, (months)**	
Time from Symptom Onset to 1^st^ medical seek	**N=138**
Median (Q1 – Q3)	5.0 (2.0 – 9.5)
Time from Symptom Onset to Rheumatologist Visit	**N=137**
Median (Q1 – Q3)	11.5 (5.0 – 20.5)
Time from Symptom Onset to Diagnosis	**N=138**
Median (Q1 – Q3)	12.8 (6.0 – 18.5)
Time from Symptom Onset to Treatment Initiation	**N=138**
Median (Q1 – Q3)	15.3 (9.0 – 23.5)
Time from 1^st^ medical seek to Rheumatologist Visit	**N=147**
Median (Q1 – Q3)	6.0 (2.0 – 12.0)
Time from 1^st^ medical seek to Diagnosis	
Median (Q1 – Q3)	6.0 (2.0 – 12.0)
Time from Diagnosis to Treatment Initiation	
Median (Q1 – Q3)	2.0 (1.0 – 3.0)

* Each participant could select more than one option. PsA: psoriatic arthritis; Q: quartile.

In the univariate models, factors associated with an increased time from symptom onset to treatment initiation included being obese, residing in suburban or rural areas instead of urban areas, having cerebrovascular diseases or hypercholesterolemia, presenting with low back pain or sleep problems as initial symptoms, and first consulting a pathologist/general practitioner, orthopaedist, or other specialists (e.g., pulmonologist, neurologist, gastroenterologist). Additionally, initial misdiagnosis was linked to delayed treatment initiation. Conversely, having an ocular autoimmune disease was associated with a decreased time to treatment initiation. In the multivariate model, being obese, residing in suburban or rural areas, having hypercholesterolemia, and experiencing an initial misdiagnosis remained significantly associated to time from symptom onset to treatment initiation (**Table 3**).

**Table 3.** Factors associated with time from symptoms onset to treatment initiation.

**Time from onset of the first symptom until treatment initiation, in months**	**Univariate models**		**Multivariate model**	
			**N=120**	
	**HR (95% CI)**	**p**	**HR (95% CI)**	**p**
**Age at diagnosis, in years**	**N=138**			
	0.99 (0.98, 1.01)	0.301		
**Sex**	**N=138**			
Female vs. Male	1.13 (0.77, 1.64)	0.531		
**BMI**	**N=138**			
Overweight vs. Underweight/Normal range	0.77 (0.52, 1.16)	**0.004**	0.86 (0.53, 1.39)	**0.011**
Obese vs. Underweight/Normal range	0.45 (0.28, 0.72)		0.45 (0.27, 0.77)	
**Residence**	**N=138**			
Suburban/rural area vs. Urban area	0.64 (0.43, 0.95)	**0.027**	0.36 (0.20, 0.63)	**<0.001**
**Education**	**N=137**			
Bachelor’s degree vs. Primary school/Lower-Upper secondary education	1.35 (0.92, 1.97)	0.087		
Master’s/Doctoral degree vs. Primary school/Lower-Upper secondary education	1.73 (0.99, 3.03)			
**Smoking habits**	**N=138**			
Former smoker vs. Smoker	1.23 (0.77, 1.96)	0.239		
No smoker vs. Smoker	1.43 (0.94, 2.16)			
**Comorbidities (Each vs. no)**	**N=129**			
Coronary heart disease	0.63 (0.35, 1.10)	0.104		
Arterial hypertension	0.72 (0.48, 1.07)	0.105		
Cerebrovascular diseases	0.17 (0.04, 0.71)	**0.015**	0.54 (0.07, 4.47)	0.569
Diabetes Mellitus	1.16 (0.70, 1.95)	0.564		
Asthma	0.89 (0.53, 1.50)	0.661		
Hypercholesterolemia	0.62 (0.43, 0.90)	**0.011**	0.61 (0.38, 0.99)	**0.046**
Osteoporosis	0.97 (0.66, 1.43)	0.892		
Crohn's disease or ulcerative colitis	1.17 (0.51, 2.72)	0.710		
Ocular disease of autoimmune etiology	2.11 (1.002, 4.46)	**0.049**	2.57 (0.98, 6.77)	0.055
Non-alcoholic fatty liver	1.29 (0.56, 3.02)	0.549		
Fibromyalgia	1.11 (0.74, 1.68)	0.607		
Depression or anxiety disorder	1.45 (0.95, 2.20)	0.083		
**Symptoms that led to seek medical care (Each vs. no)**	**N=137**			
Back Pain	0.80 (0.55, 1.18)	0.267		
Low Back Pain	0.67 (0.45, 0.998)	**0.049**	0.65 (0.37, 1.15)	0.138
Foot Problems (e.g., sole, toes, etc.)				
Joint Pain	0.71 (0.50, 1.001)	0.051		
Swollen Joints	0.94 (0.66, 1.34)	0.720		
Neck Pain	0.65 (0.41, 1.02)	0.062		
Difficulty Walking	1.22 (0.72, 2.06)	0.452		
Stiffness	0.76 (0.51, 1.14)	0.180		
Eye Problems	0.80 (0.41, 1.54)	0.497		
Fatigue	0.78 (0.54, 1.15)	0.216		
Skin Rash/Psoriasis	1.01 (0.67, 1.54)	0.945		
Tendon/Ligament Pain	0.74 (0.48, 1.15)	0.181		
Pelvic Pain	0.86 (0.12, 6.40)	0.885		
Chest Pain	0.83 (0.33, 2.06)	0.685		
Sleep Problems	0.58 (0.36, 0.95)	**0.029**	0.60 (0.33, 1.08)	0.090
Shortness of Breath	0.70 (0.28, 1.73)	0.438		
**Specialty of the physician you first visited**	**N=136**			
Pathologist/General Practitioner vs. Rheumatologist	0.46 (0.24, 0.87)	**0.022**	0.73 (0.34, 1.53)	0.507
Dermatologist vs. Rheumatologist	0.58 (0.33, 1.01)		0.62 (0.31, 1.24)	
Orthopaedist vs. Rheumatologist	0.39 (0.21, 0.73)		0.99 (0.42, 2.36)	
Other vs. Rheumatologist	0.20 (0.04, 0.92)		0.62 (0.11, 3.52)	
**Have you ever been misdiagnosed with a condition other than PsA**	**N=126**			
Yes vs. No	0.62 (0.43, 0.89)	**0.009**	0.44 (0.26, 0.74)	**0.002**

Cox regression for time-to-treatment initiation; Variables with p<0.05 were entered in the multivariate model. P-values were obtained from an overall Wald test and indicate whether each variable, as a whole, is statistically significant in the model. Bolded p-values represent statistically significant associations.

PsA: psoriatic arthritis; CI: confidence interval; HR: hazard ratio; p: p-value.

### Health-related quality of life and work productivity

**Figure 1.**
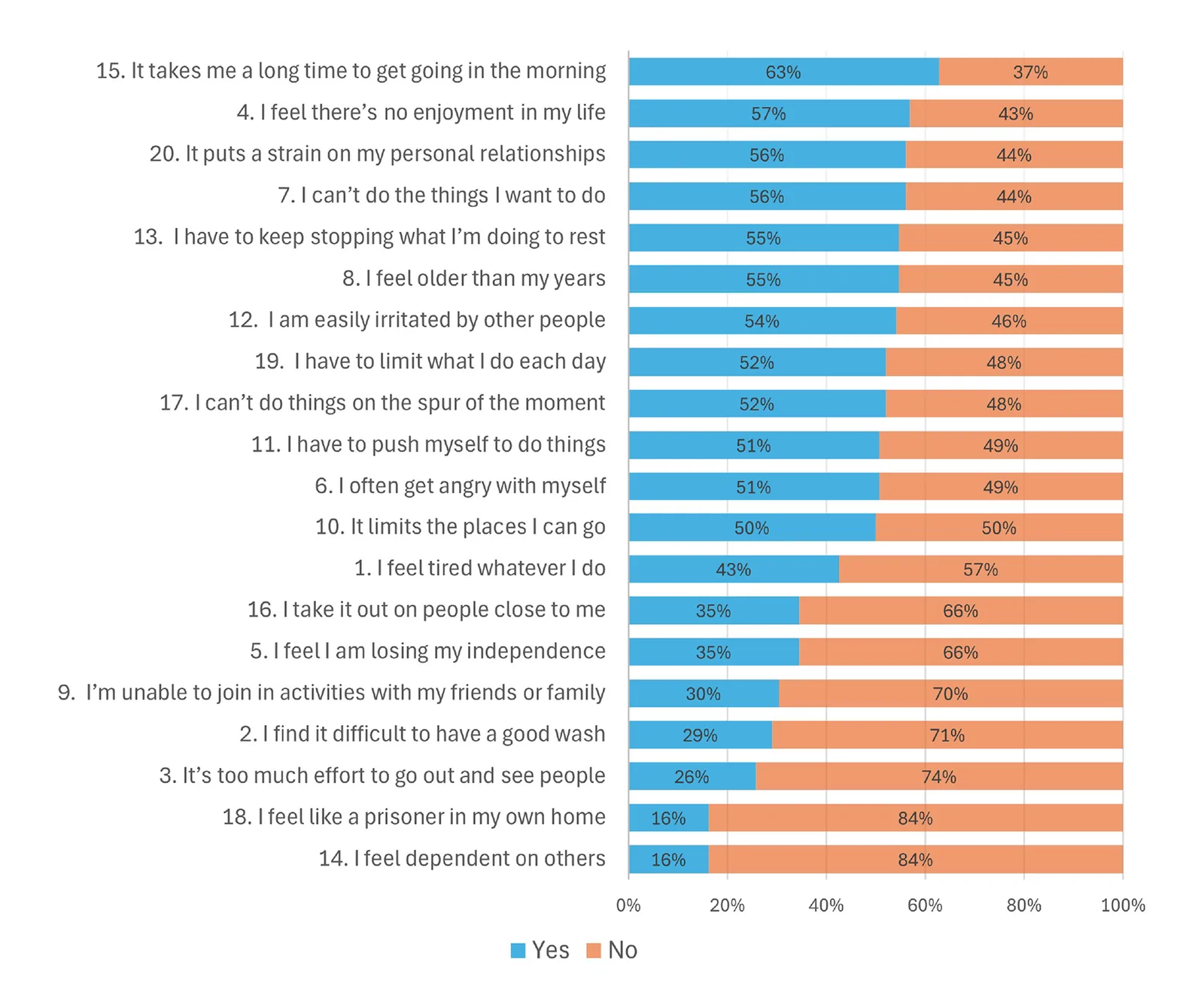
Responses to the individual Psoriatic Arthritis Quality of Life (PsAQoL) items (N=148). A horizontal bar chart illustrating the percentage of respondents who answered “Yes” (blue) or “No” (orange) to various PsAQoL statements. The highest-rated statements include “It takes me a long time to get going in the morning” (63% Yes), “I feel there’s no enjoyment in my life” (57% Yes), and “It puts a strain on my personal relationships” (56% Yes). Conversely, the least endorsed statements include “I feel like a prisoner in my own home” (16% Yes, 84% No) and “I feel dependent on others” (16% Yes, 84% No). The chart visually represents the impact of Psoriatic Arthritis on patients' physical, emotional, and social well-being.

The participants’ responses to the PsAQoL individual questions are presented in **Figure 1**. The median PsAQoL score was 9.0 (Q1: 4.0, Q3: 13.0) [mean (SD)=8.8 (5.5)]. In the univariate analysis, being female, obese, having arterial hypertension, asthma, osteoporosis, and depression or anxiety disorder were associated with poorer HRQoL. In the multivariate model, only female sex remained statistically significant (**Table 4**). Of the participants, 47% were employed at the time of the survey. Among these employed participants, the median percentage of work time missed due to PsA (absenteeism) was 0% (Q1: 0%, Q3: 5%), while the median percentage of impairment due to PsA while working (presenteeism) was 30% (Q1: 20%, Q3: 40%). The median overall work impairment due to PsA was 30% (Q1: 20%, Q3: 42%) of their total work time, and the median activity impairment was 40% (Q1: 20%, Q3: 50%) (data not shown).

**Table 4.** Factors associated with patients’ health-related quality of life.

**PsAQoL**	**Univariate models**	**p**	**Multivariate model**	**p**
			**N=137**	
	**Coef. (95% CI)**		**Coef. (95% CI)**	
**Age, in years**	**N=148**			
1-yr increase	0.03 (−0.04, 0.10)	0.386		
**Age at diagnosis, in years**				
1-yr increase	−0.02 (−0.10, 0.05)	0.537		
**Time from onset of the first symptom to diagnosis**				
1-yr increase	0.06 (−0.003, 0.12)	0.063		
**Sex**	**N=148**			
Female vs. Male	3.43 (1.57, 5.30)	**< 0.001**	2.24 (0.12, 4.36)	**0.039**
**BMI**	**N=148**			
Overweight vs. Underweight/Normal range	−0.95 (−2.93, 1.02)	**0.006**	0.52 (−1.66, 2.69)	0.382
Obese vs. Underweight/Normal range	2.67 (0.42, 4.93)		1.71 (−0.71, 4.13)	
**Smoking habits**	**N=148**			
Former smoker vs. Smoker	0.95 (−1.49, 3.38)	0.695		
No smoker vs. Smoker	0.83 (−1.34, 3.00)			
**Comorbidities (**Yes vs. No	**Ν=137**			
Coronary heart disease	0.49 (−2.39, 3.38 )	0.737		
Arterial hypertension	2.14 (0.21, 4.08)	**0.030**	0.57 (−1.48, 2.62)	0.587
Cerebrovascular diseases	0.73 (−4.08, 5.54)	0.765		
Diabetes Mellitus	0.59 (−1.92, 3.09)	0.646		
Asthma	3.23 (0.67, 5.78)	**0.013**	2.29 (−0.24, 4.82)	0.076
Hypercholesterolemia	−0.39 (−2.30, 1.52)	0.689		
Osteoporosis	3.43 (1.60, 5.27)	**< 0.001**	1.93 (−0.09, 3.94)	0.061
Crohn's disease or ulcerative colitis	−1.25 (−5.65, 3.16)	0.579		
Ocular disease of autoimmune aetiology	1.62 (−1.69, 4.93)	0.338		
Non-alcoholic fatty liver	−3.86 (−8.22, 0.50)	0.083	−2.74 (−6.85, 1.36)	0.190
Fibromyalgia	1.92 (−0.16, 4.01)	0.071	1.33 (−0.91, 3.18)	0.277
Depression or anxiety disorder	2.76 (0.70, 4.82)	**0.009**	1.80 (−0.44, 4.05)	0.115
**Current Treatment**	**N=147**			
b/tsDMARDs combinations^1^ vs. Only b/tsDMARDs	0.71 (−1.56, 2.99)	0.677		
Only csDMARDs vs. Only b/tsDMARDs	0.42 (−2.65, 3.50)			
csDMARDs combinations (excluding b/tsDMARDs)^2^ vs. Only b/tsDMARDs	1.99 (−0.71, 4.69)			
Other^3^ vs. Only b/tsDMARDs	−0.80 (−4.93, 3.34)			
No treatment vs. Only b/tsDMARDs	1.95 (−2.18, 6.09)			

1 with csDMARDs, NSAIDs, corticosteroids, and/or topical treatments;

2 with NSAIDs, corticosteroids, and/or topical treatments;

3 NSAIDs, corticosteroids, topical treatments, and/or painkillers.

GLM with gaussian family and identity link function with dependent variable the PsAQoL score (higher scores indicate poorer quality of life). Variables with p<0.15 were entered in the multivariate model. P-values were obtained from an overall Wald test and indicate whether each variable, as a whole, is statistically significant in the model. Bolded p-values represent statistically significant associations.

CI: confidence interval; Coef: coefficient; cs/b/ts DMARD: conventional/biologic/targeted synthetic disease-modifying antirheumatic drug; GLM: generalised linear model; NSAIDs: Non-Steroidal Anti-Inflammatory Drugs; PsAQoL: Psoriatic Arthritis Quality of Life; p: p-value.

### Healthcare utilisation and economic burden

In the past 12 months, 70% of participants visited a private doctor, and 6% were hospitalised, with a median hospitalisation duration of 5.0 (Q1: 3.0, Q3: 15.0) days (**Table 5**).

**Table 5.** Outpatient visits and hospitalisations during the last 12 months (N=148).

**Outpatient visits & hospitalisations last 12 months**	**N=148**
**Outpatient visits, n (%)**	
Private doctor	102 (68.9%)
Private doctor (Public sector)	10 (6.8%)
Hospital appointment (morning)	44 (29.7%)
Hospital appointment (evening)	37 (25.0%)
**Median (Q1 – Q3) number of outpatient visits/patient**	**N=102**
Private doctor (N=102)	3.0 (2.0 – 5.0)
Private doctor (Public sector) (N=10)	2.5 (0.0 – 4.0)
Hospital appointment (morning) (N=44)	6.0 (3.0 – 7.0)
Hospital appointment (evening) (N=37)	2.0 (2.0 – 3.0)
**Hospitalised, n (%)**	
Yes	9 (6.1%)
No	139 (93.9%)
**Median (Q1 – Q3) number of hospitalisations/patient**	**N=9**
Public sector	1.0 (1.0 – 3.0)
Private sector	0.0 (0.0 – 0.0)
**Median (Q1 – Q3) duration of hospitalisations, days**	5.0 (3.0 – 15.0)

Q: quartile.

The mean annual out-of-pocket expenses per patient was estimated at €609 (95% CI: €535 – €704). Treatment costs were the main driver (70%), averaging €429 (95% CI: €368 – €502), followed by outpatient visits at €178 (95% CI: €151 – €211). Visits to private doctors constituted 75% of outpatient visit costs, averaging €134 (95% CI: €111 – €165) (**Table 6**). Univariate and multivariate analysis showed that hypercholesterolemia was associated with higher out-of-pocket expenses. While residence and ocular disease did not reach statistical significance in the univariate level, both were associated with higher expenses in the multivariate level (**Table 7**). Indirect cost was estimated at €3,723 (95% CI: €2,906 – €4,859) with presenteeism cost being the main driver accounting for 82% (**Table 6**). Being younger, having coronary heart disease, arterial hypertension, osteoporosis, or ocular disease were associated with lower indirect cost, while having a Master or Doctoral degree was associated with higher indirect cost in the univariate level. All factors except education and arterial hypertension remained statistically significant in the multivariate level, where cerebrovascular diseases were also associated with lower indirect cost (**Table 7**).

**Table 6.** Annual out-of-pocket expenses and indirect cost per patient of PsA (N=146).

**Annual cost**	**Cost, Mean (95% CI)**
**Total out-of-pocket expenses/patient, €**	**609 (535 – 704)**
**Cost of outpatient visits/patient, €**	178 (151 – 211)
Private doctor	134 (111 – 165)
Private doctor (EOPYY)	4 (1 – 15)
Hospital appointment (morning)	0 (0 – 0)
Hospital appointment (evening)	40 (29 – 55)
**Cost of hospitalisations/patient, €**	3 (0 – 8)
**Cost of any treatment/patient, €**	429 (368 – 502)
**Indirect cost/patient, €**	**3,723 (2,906 – 4,859)**
Absenteeism cost, €	682 (376 – 1,568)
Presenteeism cost, €	3,041 (2,363 – 3,781)

CI: confidence interval; EOPYY: Greek National Organisation for Healthcare Services Provision.

**Table 7.** Factors associated with out-of-pocket expenses and indirect cost (Univariate and multiple models).

	**Out-of-pocket expenses**				**Indirect total cost**								

	**Univariate models**		**Multivariate model**		**Univariate models**		**Multivariate model**	

			**N=135**				**N=135**	

**Annual cost the last 12 month**s	**Coef. (95% CI)**	**p**	**Coef. (95% CI)**	**p**	**Coef. (95% CI)**	**p**	**Coef. (95% CI)**	**p**
**Age, in years**	**N=146**				**N=146**			
1-yr increase	−0.003 (−0.01, 0.01)	0.639			−0.08 (−0.12, −0.04)	**< 0.001**	−0.21 (−0.39, −0.03)	**0.025**
**Sex**	**N=146**				**N=146**			
Female vs. Male	0.15 (−0.15, 0.45)	0.319			−0.24 (−0.83, 0.35)	0.425		
**BMI**	**N=146**				**N=146**			
Overweight vs. Underweight/Normal range	−0.07 (−0.39, 0.25)	0.850			−0.44 (−1.05, 0.16)	0.344		
Obese vs. Underweight/Normal range	−0.10 (−0.46, 0.27)				−0.31 (−0.99, 0.38)			
**Residence**	**N=146**				**N=146**			
Suburban/rural area vs. Urban area	0.30 (−0.02, 0.62)	0.066	0.38 (0.05, 0.71)	**0.026**	0.02 (−0.61, 0.65)	0.962		
**Education**	**N=145**				**N=145**			
Bachelor’s degree vs. Primary school/Lower-Upper secondary education	0.09 (−0.22, 0.40)	0.840			0.55 (−0.05, 1.15)	**0.021**	0.49 (−1.58, 2.55)	0.358
Master’s/Doctoral degree vs. Primary school/Lower-Upper secondary education	0.004 (−0.45, 0.46)				1.12 (0.23, 2.00)		−2.13 (−5.12, 0.86)	
**Smoking habits**	**Ν=146**				**N=146**			
Former smoker vs. Smoker	−0.05 (−0.42, 0.32)	0.941			0.14 (−0.56, 0.84)	0.414		
No smoker vs. Smoker	0.01 (−0.33, 0.34)				−0.27 (−0.89, 0.36)			
**Comorbidities (Each vs. No)**	**N=135**				**N=135**			
Coronary heart disease	−0.21 (−0.70, 0.27)	0.390			−2.44 (−3.49, −1.39)	**< 0.001**	−5.37 (−8.79, −1.90)	**0.002**
Arterial hypertension	0.16 (−0.16, 0.47)	0.331			−1.51 (−2.33, −0.70)	**< 0.001**	−1.83 (−3.91, 0.26)	0.086
Cerebrovascular diseases	0.37 (−0.40, 1.14)	0.346			−1.35 (−2.82, 0.12)	0.072	−5.77 (−10.78, −0.77)	**0.024**
Diabetes Mellitus	0.01 (−0.39, 0.41)	0.950			−0.25 (−1.08, 0.58)	0.554		
Asthma	−0.07 (−0.48, 0.35)	0.750			−0.22 (−1.02, 0.58)	0.590		
Hypercholesterolaemia	0.33 (0.02, 0.64)	**0.039**	0.32 (0.01, 0.62)	**0.040**	−0.38 (−1,04, 0.26)	0.239		
Osteoporosis	0.24 (−0.08, 0.56)	0.139	0.11 (−0.20, 0.42)	0.488	−0. 94 (−1.56, −0.32)	**0.003**	−2.87 (−5.04, −0.71)	**0.009**
Crohn's disease or ulcerative colitis	−0.58 (−1.27, 0.11)	0.101	−0.45 (−1.12, 0.23)	0.194	1.03 (−0.33, 2.89)	0.139	2.07 (−1.63, 5.76)	0.273
Ocular disease of autoimmune aetiology	0.48 (−0.05, 1.01)	0.079	0.57 (0.06, 1.08)	**0.027**	−1.31 (−2.38, −0.25)	**0.016**	−6.42 (−11.32, −1.52)	**0.010**
Non-alcoholic fatty liver	−0.45 (−1.15, 0.25)	0.206			−0.78 (−2.11, 0.54)	0.246		
Fibromyalgia	0.21 (−0.14, 0.55)	0.241			−0.15 (−0.79, 0.49)	0.646		
Depression or anxiety disorder	0.11 (−0.23, 0.46)	0.509			−0.005 (−0.65, 0.65)	0.989		
**Current Treatment**	**N=145**				**N=145**			
b/tsDMARDs combinations^1^ vs. Only b/tsDMARDs	0.30 (−0.06, 0.65)	0.494			−0.39 (−1.05, 0.26)	0.723		
Only csDMARDs vs. Only b/tsDMARDs	0.16 (−0.32, 0.64)				−0.39 (−1.29, 0.51)			
csDMARDs combinations (excluding b/tsDMARDs)^2^ vs. Only b/tsDMARDs	0.16 (−0.25, 0.58)				−0.05 (−0.82, 0.72)			
Other^3^ vs. Only b/tsDMARDs	−0.15 (−0.79,0.48)				−0.61 (−1.79, 0.58)			
No treatment vs. Only b/tsDMARDs	0.39 (−0.25, 1.02)				0.02 (−1.16, 1.20)			

1 with csDMARDs, NSAIDs, corticosteroids, and/or topical treatments;

2 with NSAIDs, corticosteroids, and/or topical treatments;

3 NSAIDs, corticosteroids, topical treatments and/or painkillers.

GLM with gamma family and a log link function. All variables with p < 0.15 in the univariate model, were inserted to the multivariate model. P-values were obtained from an overall Wald test and indicate whether each variable, as a whole, is statistically significant in the model. Bolded p-values represent statistically significant associations.

CI: confidence interval; Coef: coefficient; cs/b/ts DMARD: conventional/biologic/targeted synthetic disease-modifying antirheumatic drug; GLM: generalised linear model; NSAIDs: Non-Steroidal Anti-Inflammatory Drugs.

## DISCUSSION

To the best of our knowledge, this is the first comprehensive attempt to explore the patient journey and assess the impact of PsA on HRQoL, work impairment and out-of-pocket expenditures in Greece. Our results regarding diagnostic delays are comparable with patterns observed in the literature. Patients experienced a median time of 5.0 months from symptom onset to their first medical consultation, while the median time to rheumatologist consultation was 11.5 months, and to diagnosis was 12.8 months. The LOOP study reported shorter delays from symptoms’ onset to PsA diagnosis in Eastern Europe and the Middle East (3.0 months), and Western Europe (5.0 months), while the Swiss Clinical Quality Management (SCQM) registry highlighted considerable diagnostic delays, higher from our findings, showing a mean diagnostic delay of 4.5 years for axial PsA and 3.2 years for peripheral PsA, underscoring the need for improved diagnostic strategies.^29,30^ Findings for slightly lower delays compared to ours are reported for Japan and Asia-Pacific, while findings from the Brazilian sub-dataset of the LOOP study reported significant higher results.^29,31^ The hazard ratio (HR) of 0.58 (95% CI: 0.33, 1.01) for the time from onset of the first symptom until treatment initiation, comparing dermatologists to rheumatologists in our study, suggests a trend towards quicker treatment initiation when managed by rheumatologists found elsewhere, although this did not reach statistical significance.^31^

The mean (SD) and median (Q1 – Q3) PsAQoL score in our cohort was 8.8 (5.5) and 9.0 (4.0 – 13.0), respectively. Additionally, 42.6% of our patients were classified as having fatigue according to the 1^st^ item of PsAQoL. This is comparable to data from other studies, such as the Utah Psoriasis Initiative Arthritis registry, which reported that 50.5% of participants were classified as having fatigue according to the 1^st^ item of PsAQoL.^32^ Another cross-sectional study, conducted in hospital patients in Madrid, reported a mean PsAQoL score of 6.6 (SD: 5.7), demonstrating the considerable burden of PsA on patients’ quality of life.^33^ Additionally, data from the Adelphi PsA Disease Specific Programme (DSP), which surveyed patients in multiple countries, indicated that women reported worse HRQoL than men, using a different instrument. In our study, the difference in HRQoL between females and males was also significant, with a coefficient (95% CI) of 2.20 (0.22, 4.19) in PsAQoL.^34^

Our findings on work productivity and activity impairment among patients with PsA highlight the substantial impact of the disease on daily functioning and employment. Our findings align with data from other studies. For instance, a study from the Netherlands reported a median (IQR) absenteeism of 0% (0%–0%), presenteeism of 20% (0%–40%), overall work impairment of 10% (0%–40%), and activity impairment of 30% (10%– 60%).^35^ Additionally, the Brazilian sub-cohort from LOOP study reported a mean (SD) overall work impairment score of 10.7 ± 19.1 among patients with PsA.^31^ The main LOOP cohort, reported a mean (SD) overall work impairment score of 30.1 (30.2) for rheumatologists, and 29.5 (31.7) for dermatologists, and a mean (SD) activity impairment score of 35.0 (29.8) for rheumatologists, and 7.6 (7.2) for dermatologists.^29^ A large-scale cross-sectional survey conducted with rheumatologists and dermatologists and their patients with PsA across Australia, Canada, France, Germany, Italy, Japan, Spain, UK, and the US further confirmed these findings, showing a median (IQR) percent work time missed due to PsA of 0% (0%–0%), percent impairment while working of 20% (0%–30%), overall work impairment of 20% (0%–30%), and activity impairment of 20% (10%–40%).^36^

In a systematic literature review published in 2015, the annual indirect cost of PsA with the friction method in 4 European countries ranged from US $2,053 to $3,716 (in 2015 values). Accounting for inflation and the exchange rate of USD to EUR, these results are comparable with our findings of €3,723.^37^ In a recent Colombian study (2021), the annual out-of-pocket expenses for medication, medical appointments, and examinations were approximately 160 USD (in 2019 values), which is lower than our findings.^38^ However, a larger study from the USA in the same year reported out-of-pocket expenses ranging from $4,423 to $6,950.^39^ It is important to note that these findings are not directly comparable to ours, as the U.S. study focused on expenses for biologic treatments, which are fully reimbursed in Greece.

This study’s primary strength lies in its comprehensive analysis of experiences of patients with PsA, covering aspects from comorbidities to economic burden, which provides valuable insights into the multifaceted challenges faced by these patients. The adequate sample size and detailed questionnaire allowed for an in-depth understanding of the disease’s impact on quality of life, work productivity, and healthcare utilisation. However, several limitations should be noted. The cross-sectional design limits the ability to establish causal relationships between variables. The use of an online survey may have introduced selection bias, excluding individuals without internet access or those less tech-savvy, potentially skewing the results. Recall bias is another concern, as the data relies on self-reported information from participants, particularly regarding medical history and treatment timelines. It should also be acknowledged that disease activity was not assessed or collected in any form, whether through clinical examination, laboratory testing, or patient-reported measures. This decision was intentional, as the primary aim of the study was to explore the patient journey and self-perceived burden of PsA, rather than to evaluate disease activity or treatment response. While this patient-centred approach offers valuable insights into real-world experiences, the absence of objective or standardised disease activity measures may introduce subjectivity and potential confounding in the interpretation of associations. The study’s focus on members of a single patient association might limit the generalisability of the findings to the broader PsA population. It should also be noted that the study sample predominantly consisted of female participants (70%), which may affect the generalisability. This imbalance likely reflects the characteristics of the patient association membership, as female patients are often more likely to participate in survey-based or patient-organisation research initiatives. Seasonal variations in disease symptoms and healthcare utilisation, given the eight-month data collection period, may also have influenced the results.

This study offers valuable and up-to-date insights into the diagnostic delays and the humanistic and economic burdens faced by patients with PsA in Greece, addressing a gap in the existing literature. These findings can support healthcare providers and policymakers in making more informed decisions to improve patient care and resource allocation.

## Data Availability

The datasets generated and/or analysed during the current study are not publicly available but are available from the corresponding author upon reasonable request.
